# Cumulative Effects of Low Impact Development on Watershed Hydrology in a Mixed Land-Cover System

**DOI:** 10.3390/w10080991

**Published:** 2018-07-27

**Authors:** Nahal Hoghooghi, Heather E. Golden, Brian P. Bledsoe, Bradley L. Barnhart, Allen F. Brookes, Kevin S. Djang, Jonathan J. Halama, Robert B. McKane, Christopher T. Nietch, Paul P. Pettus

**Affiliations:** 1 Oak Ridge Institute for Science and Education, c/o US Environmental Protection Agency, Office of Research and Development, National Exposure Research Laboratory, Cincinnati, OH 45268, USA; 2 Institute for Resilient Infrastructure Systems, College of Engineering, University of Georgia, Athens, GA 30602, USA; 3 National Exposure Research Laboratory, Office of Research and Development, US Environmental Protection Agency, Cincinnati, OH 45268, USA; 4 Western Ecology Division, National Health and Environmental Effects Research Laboratory, US Environmental Protection Agency, Corvallis, OR 97330, USA; 5 Inoventures LLC, Western Ecology Division, National Health and Environmental Effects Research Laboratory, c/o US Environmental Protection Agency, Corvallis, OR 97330, USA; 6 National Risk Management Research Laboratory, US Environmental Protection Agency, Cincinnati, OH 45268, USA

**Keywords:** LID practices, watershed scale, impervious area, peak flow, surface runoff, shallow subsurface runoff and infiltration, evapotranspiration

## Abstract

Low Impact Development (LID) is an alternative to conventional urban stormwater management practices, which aims at mitigating the impacts of urbanization on water quantity and quality. Plot and local scale studies provide evidence of LID effectiveness; however, little is known about the overall watershed scale influence of LID practices. This is particularly true in watersheds with a land cover that is more diverse than that of urban or suburban classifications alone. We address this watershed-scale gap by assessing the effects of three common LID practices (rain gardens, permeable pavement, and riparian buffers) on the hydrology of a 0.94 km^2^ mixed land cover watershed. We used a spatially-explicit ecohydrological model, called Visualizing Ecosystems for Land Management Assessments (VELMA), to compare changes in watershed hydrologic responses before and after the implementation of LID practices. For the LID scenarios, we examined different spatial configurations, using 25%, 50%, 75% and 100% implementation extents, to convert sidewalks into rain gardens, and parking lots and driveways into permeable pavement. We further applied 20 m and 40 m riparian buffers along streams that were adjacent to agricultural land cover. The results showed overall increases in shallow subsurface runoff and infiltration, as well as evapotranspiration, and decreases in peak flows and surface runoff across all types and configurations of LID. Among individual LID practices, rain gardens had the greatest influence on each component of the overall watershed water balance. As anticipated, the combination of LID practices at the highest implementation level resulted in the most substantial changes to the overall watershed hydrology. It is notable that all hydrological changes from the LID implementation, ranging from 0.01 to 0.06 km^2^ across the study watershed, were modest, which suggests a potentially limited efficacy of LID practices in mixed land cover watersheds.

## 1. Introduction

Urbanization alters natural hydrological systems by altering stream channel networks (e.g., channelization and burial), creating microclimates (e.g., urban heat islands), and generating rapid runoff from precipitation and snowmelt events [1]. These changes have direct impacts on surface and groundwater quantity and quality. Conventional urban stormwater management practices are often developed to control runoff and minimize flooding; however, these systems can be costly and may not directly address issues, such as reductions in infiltration and groundwater storage via impervious surfaces that may lead to urban flooding, erosion, and the degradation of water quality [2].

In recent years, alternative stormwater management practices, such as Low Impact Development (LID), have been adopted (e.g., bioretention cells or rain gardens, permeable pavements, and bioswales) in many urban and suburban areas [3,4]. LID, also called sustainable urban drainage systems (SUDSs), among other globally varying names [5], is an approach that uses soils, vegetation, and landscape design to control nonpoint source runoff and pollutants in urban systems. A goal of LID is to promote watershed resilience through “green” design [6].

There is a growing body of literature focused on evaluating the local (e.g., plot or site) scale effectiveness of LID. Several recent papers have synthesized the key findings of studies assessing the effects of different LID practices, including field experiments and modeling studies, on water quantity (e.g., peak flow and runoff volume) and water quality (e.g., nitrogen, phosphorous, and total suspended solids) at local scales [7–11]. These previous studies provide foundational research for scaling LID approaches to watersheds. However, limited evidence of LID effectiveness at the watershed scale exists [12,13], and research focusing on LID impacts at watershed scales is just beginning to emerge [14]. Therefore, questions remain about how LID practices can individually or cumulatively affect watershed hydrology [14,15].

Experimental studies, designed to investigate the watershed-scale effects of LID, have provided critical insights into how watershed hydrology responds to these approaches. For example, Jarden et al. [16] designed a paired watershed approach to quantify the effect of street-connected bioretention cells, rain gardens, and rain barrels on peak discharge and total storm runoff. The results from the subwatershed with smaller LID lots and underdrain connections showed a substantial reduction in peak discharge (up to 33%) and total storm runoff (up to 40%). Additionally, recent field-based research provides evidence of the cumulative watershed scale effects of LID on hydrologic responses, such as peak flows and pollutant loads [17–20]. Such experimental studies can be resource intensive (e.g., financial, personnel, time) [21]; however, process-based models provide a means to go beyond measured data and explore the projected “what if” LID scenarios using potentially less resources.

Process-based or mechanistic watershed models, which simulate hydrological (and other) processes and outputs for different water balance components (e.g., streamflow, evapotranspiration), are critical tools to understand the influence of LID practices on watershed processes [22]. The Storm Water Management Model (SWMM) [23] has been used to simulate the effects of different LID practice implementations (porous pavement, rain barrels, and rain garden) on runoff and flood risk reductions in an 87.6 km^2^ urban watershed and model the cumulative effects of street-side bioretention cells, rain gardens, and rain barrels in a 0.12 km^2^ residential watershed [21]. Overall, the results indicate increases in evaporation and infiltration, as well as decreases in surface runoff and discharge, across different return periods. Tsshe performance of LID practices (rain gardens, permeable pavements, and rainwater harvesting tanks) has also been evaluated under different urban land use densities using the Soil and Water Assessment Tool (SWAT) [24], demonstrating that the effectiveness of LID practices differs among the urban land use densities [25].

The aforementioned studies and others (e.g., [26–29]) advance current knowledge on the effectiveness of LID practices at watershed scales using various process-based model approaches; however, all of these studies focus on watersheds that are entirely urban or suburban. A clear need exists for an understanding of the extent to which LID approaches are effective in mixed land cover watersheds, i.e., those with urban and suburban land cover *in addition to others* (e.g., forest and agriculture). Furthermore, a spatially explicit approach toward representing LID practices and associated hydrological processes to analyze the effects of varying patterns of mixed land use and land cover under different management practices is critical [30], as most approaches are challenged with representing spatial landscape heterogeneities [31].

In this paper, we assess how LID implementation affects watershed hydrologic responses in a mixed land cover watershed. Specifically, we ask: How does the type and extent of LID practices affect water balance components, including surface runoff, peak flows, evapotranspiration, shallow subsurface flow, and infiltration, in a mixed land cover watershed? We do this by using a spatially-explicit ecohydrological model, called Visualizing Ecosystems for Land Management Assessments (VELMA) [32] for a variety of scenarios associated with LID and the implementation of forested riparian buffers. Our study is one of the first, to our knowledge, to examine LID implementation at the watershed scale using spatially explicit modeling approaches in a system with mixed suburban, agricultural and forest land cover. As a result, we discuss the implications of this study for effective stormwater management in mixed land cover systems and future research directions toward this goal.

## 2. Materials and Methods

### 2.1. Study Area Description

**Figure 1:**
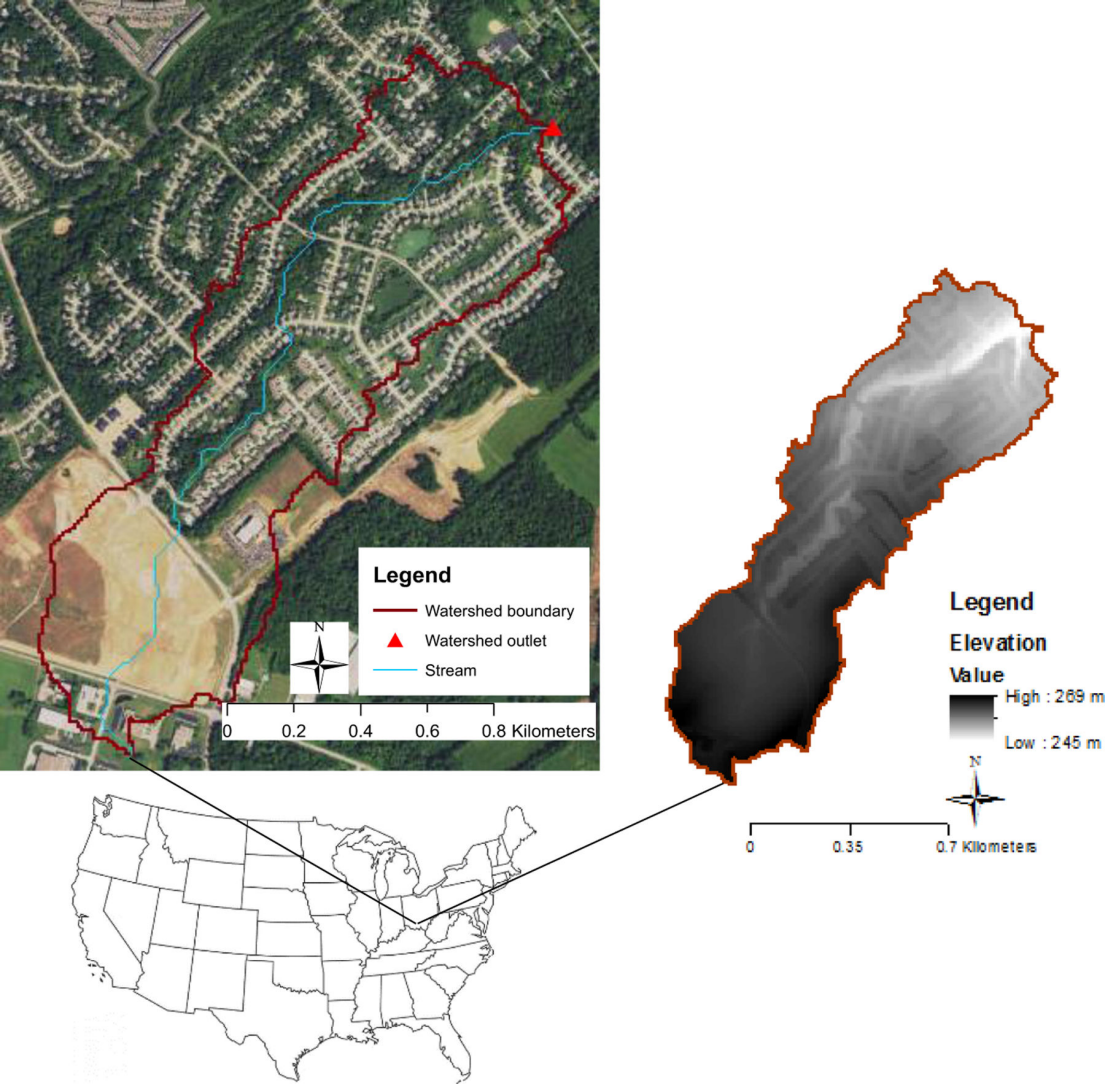
The study area is a 0.92 km^2^ subwatershed (Shayler Crossing) of the East Fork Little Miami River watershed, located on the east side of Cincinnati, Ohio in Clermont County, USA. The watershed outlet is identified as a red triangle.

The Shayler Crossing (SHC) watershed is a subwatershed of the East Fork Little Miami River Watershed in southwest Ohio, USA and falls within the Till Plains region of the Central Lowland physiographic province. The Till Plains region is a topographically young and extensive flat plain, with many areas remaining undissected by even the smallest stream. The bedrock is buried under a mantle of glacial drift 3–15 m thick [33,34]. The Digital Elevation Model (DEM) has a maximum value of ~269 m (North American_1983 datum) within the watershed boundary (Figure 1). The soils are primarily the Avonburg and Rossmoyne series, with high silty clay loam content and poor to moderate infiltration [35]. Average annual precipitation for the period, 1990 through 2011, was 1097.4 *±* 173.5 mm. Average annual air temperature for the same period was 12 *°*C [36].

We considered SHC a mixed land cover watershed, located on the east side of Cincinnati, Ohio, with a drainage area of 0.92 km^2^ (Figure 1). The primary land uses consist of 64.1% urban or developed area (including 37% lawn, 12% building, 6.5% street, 6.4% sidewalk, and 2.1% parking lot and driveway), 23% agriculture, and 13% deciduous forest (Table 1). Total imperviousness covers approximately 27% of the watershed area, the majority of which is directly connected to a storm sewer system without any intermediary [30]. The watershed was chosen for this study because it is part of the East Fork Little Miami River Watershed, where a long-term monitoring and focused modeling effort is being conducted by the US Environmental Protection Agency (EPA), Office of Research and Development (ORD), Ohio Environmental Protection Agency (Ohio EPA), and Clermont County (Ohio) Stormwater Division.

### 2.2. Input Data

We obtained average daily precipitation and temperature data from a weather station, located approximately 13 km from the north boundary of the watershed at 84.2909° W, 39.194° N [37]. Streamflow has been monitored, from 3 April 2006 to the present day, using a stage sensor (600 LS Sonde with temperature, conductivity, and shallow vented level sensors, YSI Inc., Yellow Springs, OH, USA) at the watershed outlet. Water depth was recorded at 10-min intervals and converted to streamflow (m^3^ *s*^−1^) using a rating curve, developed by US EPA. We obtained a 10 m resolution DEM, Soil Survey Spatial Tabular (SSURGO 2.2) soil data, and National Land Cover Dataset (NLCD) land use data from the Natural Resources Conservation Service (NRCS) Geospatial Data Gateway [38]. We further used an impervious area shape file from Clermont County, Ohio through the Center for Urban Green Infrastructure Engineering, Inc. (Milford, OH, USA).

### 2.3. Model Description

To simulate the effect of LID on watershed hydrology, we used the Visualizing Ecosystems for Land Management Assessments (VELMA) model. VELMA is a spatially distributed ecohydrological model that couples watershed hydrology and carbon (C) and nitrogen (N) cycling in plants and soils, and the transport of water, C, and N from the terrestrial landscape to streams [32]. VELMA is not an “urban hydrology” model according to the strict tradition of stormwater management models (e.g., SWMM). Its key strengths are its spatially explicit representation of hydrological and biogeochemical processes and broad applicability to a variety of ecosystems, such as forest, agricultural, and urban, in order to assess the effects of LID in mixed land cover systems. Urban LID practices can be represented in the model using modifications to present watershed permeability, lateral and horizontal hydraulic conductivities, and land cover (see Section 2.5). VELMA’s spatially explicit grid-based structure affords the capacity to represent transitions from directly connected to indirectly connected impervious areas by replacing values on a cell by the cell basis for the aforementioned model representations. The model is also capable of scaling hydrologic and biogeochemistry responses across multiple spatial (hillslopes to basins) and temporal (days to centuries) scales [21]. VELMA’s visualization and interactivity features are packaged in an open-source, open-platform programming environment (Java/Eclipse) [32].

VELMA’s modeling domain is a three-dimensional matrix that includes information regarding surface topography, land use, and four soil layers. VELMA uses a distributed soil column framework to model the lateral and vertical movement of water and nutrients through the four soil layers. A soil water balance is solved for each layer. The soil column model has three coupled submodels: (1) A hydrological model that simulates the vertical and lateral movement of water within the soil and losses of water from soil and vegetation in the atmosphere; (2) a soil temperature model that simulates daily soil layer temperatures based on surface air temperature; and (3) a biogeochemistry model that simulates C and N dynamics.

A simple logistical function, based on the degree of saturation, is applied to capture the breakthrough characteristic of soil water. Potential evapotranspiration (PET) is estimated using the simple temperature-based method of Hamon [39]. Evapotranspiration (ET) increases exponentially as soil water storage increases, and it reaches the PET rate as the soil water storage reaches saturation. The VELMA simulator engine allows for the specification of a spatial data map, with permeability fractions for each grid cell value (here, each 10 m grid cell). The grid’s permeability fractions are taken into account when determining how much of a cell’s total water inflow (e.g., from rain, snow melt, and lateral surface movement) penetrates into the first layer of the soil column. A permeability of 0 is completely impermeable (no water penetrates from the surface to the first soil layer), and 1 is completely permeable (all water penetrates from the surface to the first soil layer).

**Figure 2:**
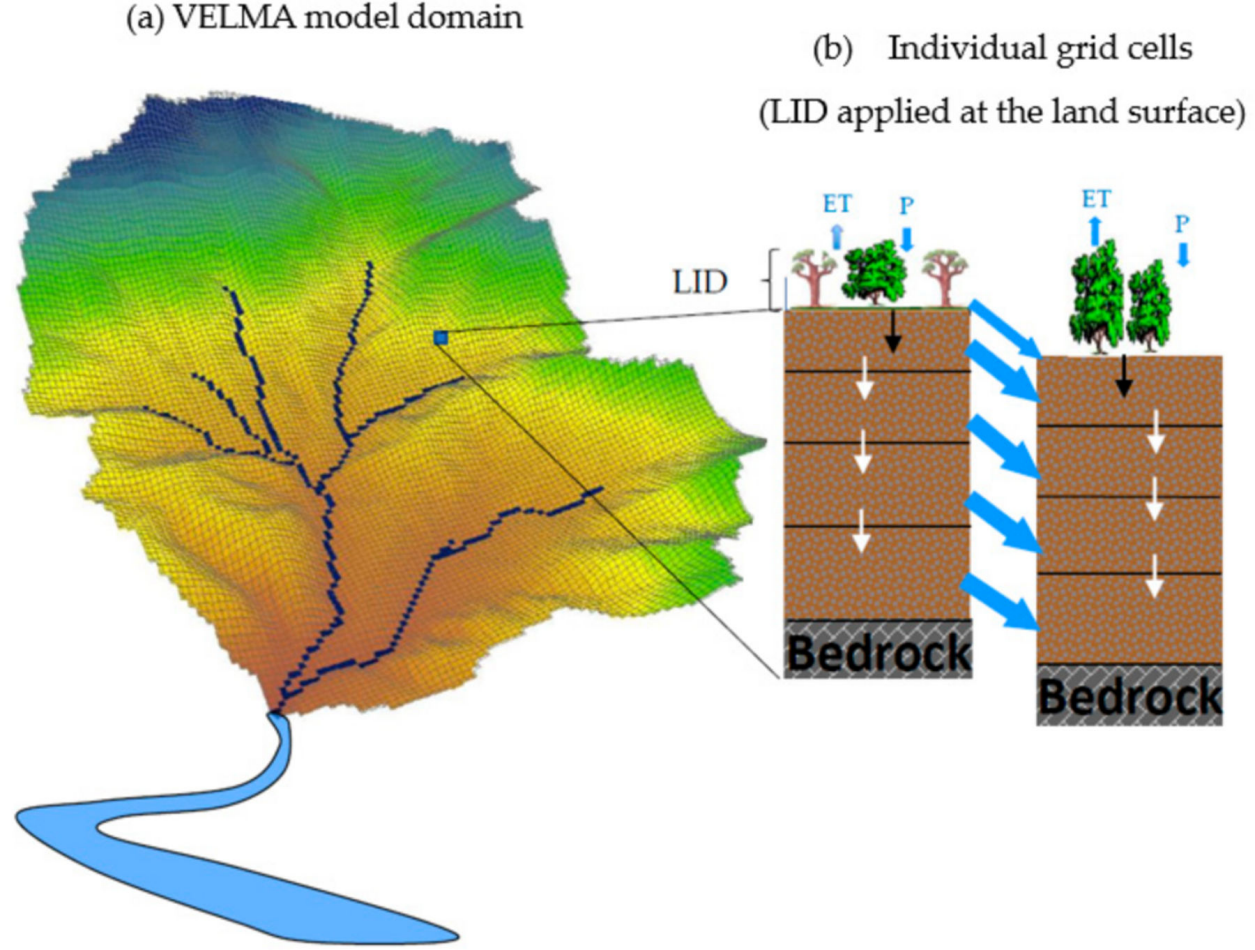
Generalized structure of the VELMA model, and applications for LID. VELMA’s domain is a watershed (**a**). Each grid cell within the watershed has four soil layers (**b**), including LID applied to the land surface). Infiltration from LID is transported to the first soil layer (black arrow). Further vertical transport of water (in this paper), carbon, and nitrogen transport can occur between each grid cell’s four layers (white arrows), and surplus water (or carbon, nitrogen) is transferred from a grid cell to the adjacent, most down gradient cell(s) in the watershed (blue arrows). P = Precipitation; ET = evapotranspiration. Modified from Abdelnour et al. [21].

The soil column model is placed within a watershed framework to create a spatially distributed model applicable to watersheds (Figure 2, shown here with LID practices). Adjacent soil columns interact through down-gradient water transport. Water entering each pixel (via precipitation or flow from an adjacent pixel) can either first infiltrate into the implemented LID and the top soil layer, and then to the downslope pixel, or continue its downslope movement as the lateral surface flow. Surface and subsurface lateral flow are routed using a multiple flow direction method, as described in Abdelnour et al. [21]. A detailed description of the processes and equations can be found in McKane et al. [32], Abdelnour et al. [21], Abdelnour et al. [40].

### 2.4. Watershed Model Setup

We used VELMA’s pre-processor tool (called Java Processing Digital Elevation Model (JPDEM) [32,41]) to fill sinks, determine flow direction, and compute the flow contribution area of a 10 m DEM [32]. The watershed boundary was delineated, and the watershed outlet was assigned using VELMA’s pre-processor [32]. All DEM, soil, and land use maps were clipped so that they have the same number of columns and rows for the American Standard Code for Information Interchange (ASCII grid, Esri, Inc., Esri grid format ArcGIS Desktop 10.0 Help, http://desktop.arcgis.com/en/arcmap/10.3/manage-data/raster-and-images/esri-grid-format.htm) input in VELMA. The soil and the land use maps contained ID numbers for every cell in the simulation area, which corresponded to one or more of VELMA’s simulator configurations. We assigned two of VELMA’s soil configurations to represent Rossymoyne and Avonburg soil types and seven land use configurations to represent agriculture, forest, lawn, buildings, streets, sidewalks, parking lots and driveways. We merged wet pond pixels with lawn pixels because currently lakes and ponds are not implementable in VELMA.

### 2.5. Base Model Parameterization, Calibration and Validation

We performed the base model calibration with daily streamflow at the outlet of the watershed from 1 January 2009 to 31 December 2010 with 2008 as a model warmup period and from 1 January 2011 to 31 December 2011 as a validation period. Our calibration period (2009 and 2010) included normal precipitation years (1040 and 1046 mm), and our verification period (2011) was a wet year (1660 mm). We defined a ‘wet’ period as greater than one standard deviation from the mean precipitation (>1270.1 mm) and a dry period as less than one standard deviation (<923.9 mm).

Calibration was conducted through both semi-automatic and manual calibrations. We used autocalibration to screen for sensitive parameters and reduce the solution space. Manual calibration was implemented as a second phase to further refine the parameter values. For the initial automatic calibration, we used the MOEA-VELMA calibration tool that links VELMA with the Multiobjective Evolutionary Algorithm (MOEA) [42] framework in Java. The MOEA framework uses evolutionary algorithms to solve multiobjective optimization problems, and the MOEA-VELMA calibration tool leverages this ability to tune model input parameters to minimize the differences between simulated results and observed data. Several parameters were chosen to calibrate the model, including soil layer thickness, saturated hydraulic conductivity, porosity fraction, bulk density, wilting point, field capacity, and PET parameters. The MOEA-VELMA calibration tool then implemented NSGA-II [43], using the MOEA framework, and searched for the optimal set of input parameters to optimize our objective function, that is, Nash Sutcliffe Efficiency (NSE) [44] for the observed and predicted daily streamflow:

(1) $$NSE=1-\frac{\sum_{i=1}^{n}{\left|O_{i}-P_{i}\right|}^{2}}{\sum_{i=1}^{n}{\left|O_{i}-\bar{O}\right|}^{2}}$$

where *O_i_* is the ith measured variable (e.g., discharge), *P_i_* is the ith predicted variable, $\bar{O}$ is the arithmetic average of the measured variable, and *n* is the total number of observations. The NSE coefficient ranges between 1 (perfect fit) and negative infinity. An efficiency below zero implies that the mean value of the observed value is a better predictor than the model.

After almost 500 simulations, we narrowed the range of selected sensitive parameters and ran the MOEA-VELMA calibration tool for an additional 500 simulations. Then, we picked the solutions with a higher NSE and used those parameter ranges in the manual calibration.

After the initial semi-automatic calibration, we conducted manual calibration through visual analysis to capture trends in observed streamflow, using NSE in addition to percent bias (*PBIAS*) [45] and root mean squared error (RMSE) [46]. PBIAS measures the average tendency of the predicted data to be larger or smaller than observed values. It is also measures over- and underestimation of bias [44]:

(2) $$PBIAS=\frac{\sum_{i=1}^{n}\left(O_{i}-P_{i}\right)}{\sum_{i=1}^{n}O_{i}}\;\;\times \;100$$

and RMSE is the square root of the mean square error and varies from zero to large positive values:

(3) $$RMSE=\sqrt{\frac{1}{n}\sum_{i=1}^{n}{\left(O_{i}-P_{i}\right)}^{2}}$$

To ensure the simulations provided reasonable volumetric matches with observed data, we also used a total simulated to total observed annual streamflow ratio (Sim:Obs) for each simulation year. If the Sim:Obs was >1, simulated streamflow from the year exceeded that of the observed streamflow. If it was <1, the opposite was true, and Sim:Obs = 1 suggested a perfect match between the total annual simulated and observed streamflow.

The soil thickness of each layer was parameterized using United States Department of Agriculture (USDA) soil survey data for the study area [35]. We used the MOEA-VELMA calibration tool to calibrate saturated vertical and horizontal hydraulic conductivities for each soil layer. Other soil physical characteristics (porosity, field capacity, wilting point, and bulk density) were obtained based on soil texture class (Table 2) [32]. We obtained the first term of the PET *Hamon* equation (petParam1) for different cover types using the MOEA-VELMA calibration tool, with the second term of *Hamon* equation set to a constant value of 0.622, based on Abdelnour et al. [21]. A *be* parameter is a calibration constant; it is an ET coefficient used in the logistic equation that computes ET from PET. We estimated this parameter value from autocalibration. Air density (*roair*) was constant and set to 1300 g m^*−*3^ (Table 3). We adjusted all soil physical characteristics and PET parameters to best match the observed streamflow during manual calibration. The parameters and their final model values are shown in Tables 2 and 3. Setting soil parameters to zero produces an error in the VELMA output; therefore, we set soil parameter values for impervious areas to those of the clay soil texture class, using the approach by McKane et al. [32].

### 2.6. Low Impact Development (LID) Configurations, Scenarios, and Model Parameters

To evaluate the effectiveness of LID practices based on the relative daily changes in watershed hydrology compared to the calibrated base model, we simulated three types of LID scenarios: Rain gardens (RG), permeable pavements (PP), and forested riparian buffers (RB). Our goal was to derive a relative understanding of how different spatial distributions of select LID types may affect hydrology in this mixed land over system; therefore, we did not aim to represent specific stakeholder-selected LID practices for the watershed (e.g., exact sites where landowners would agree to implementation).

**Figure 3:**
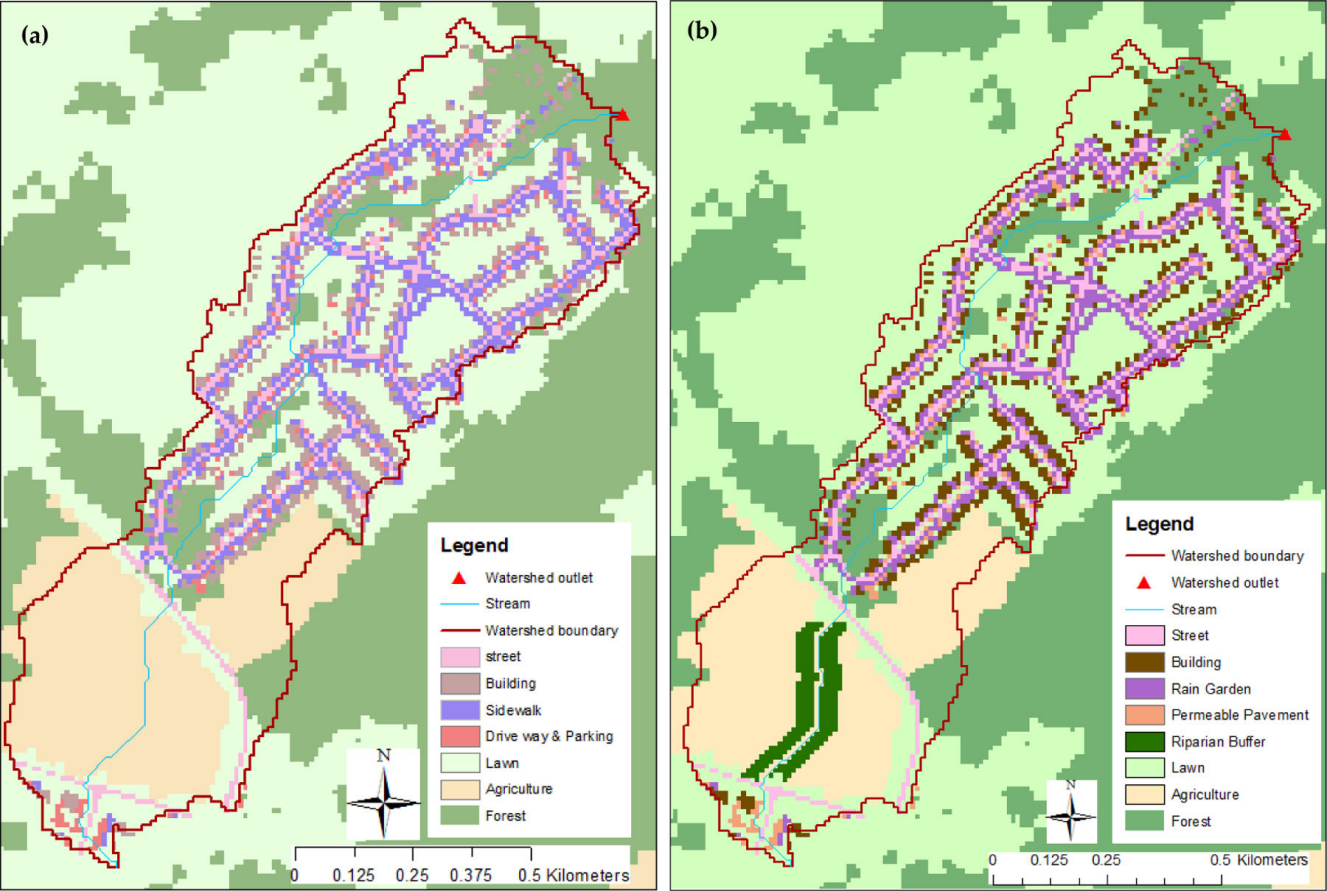
The spatial configuration of different land covers in the SHC watershed, with 10m cell resolution: (**a**) Current land cover, and (**b**) after LID implementation: Conversion of a 100% spatial configuration of the sidewalks to rain gardens, and driveways and parking lots into permeable pavements, and the implementation of 40 m forest buffers along both sides of the stream on the agricultural land. The watershed outlet is identified as a red triangle.

To implement the LID scenarios, we replaced grid cells in the calibrated base model, identified as impervious, areas with one of two LID practices: RG or PP, depending on the impervious area type (see below). We further replaced grid cells in agricultural land cover along a stream with RB. We ran each scenario as a separate model using evenly distributed spatial configurations of 25%, 50%, 75% and 100% conversions for RG (in sidewalk locations) and PP (at parking lots and driveways; see Figure 3 for an example). Each spatial distribution of RG and PP met or exceeded the watershed’s water quality volume for bioretention (i.e., generally speaking, the volume of water treated by LID practices to control in low to medium magnitude storm events), as recommended by the Ohio Department of Natural Resources [47]. We also placed RP at 20 m and 40 m on each side of the stream in the agricultural land of the Northern part of the watershed (Figure 1). This resulted in 10 simulated LID scenarios (4 RG, 4 PP, and 2 RB) for comparison. We note here that a large-scale conversion of impervious areas to LIDs (e.g., our 100% conversion scenarios) may not be reasonable, in terms of both financial cost and the willingness of the community [46]; however, these conversion configurations can provide a maximum mitigation potential for decision support.

RG and PP were chosen for the scenarios because they are reasonable retrofitting measures for the studied watershed, are the most promising LID practices for reductions in peak flow and runoff volume [8,48], and can be applied and assessed in the VELMA model. RBs were selected because they currently do not exist in the agricultural land of the watershed (and therefore the base model). Their addition was used for comparisons of the watershed-scale hydrological responses of RG and RB conversions on impermeable areas. We selected 20 m and 40 m buffers to go beyond Ohio EPA’s requirement that forested area must be maintained for a minimum of the first 15 m of the area on either bank [49].

We assessed the effect of the LID scenario implementation: (a) Individually and (b) using an LID combination scenario (i.e., fully implementing RG, PP, and RB with the maximum level of implementation). The individual model scenario runs of land cover conversions to RG and PP, for each spatial configuration, included: (a) Sidewalks were converted to RG and (b) parking lots and driveways were converted to PP (Table 4 and Figure 3). Lawns were not converted to RG. The percentage of the watershed that was converted to RG, PP, and RB practices at different implementation levels is shown in Table 5.

To implement LID into each scenario, we parameterized the soil texture, soil physical characteristics, and PET parameter values for LID practices, based on Ohio EPA requirements (Table 6) [49]. To do this for the RG scenarios, we created soil maps with a new soil class, “RG,” for each spatial configuration (i.e., 25% to 100%). The RG soil maps, one for each implementation level, replaced the sidewalk pixels of the original soil map. Soils in the new RG maps were adjusted for soil depths, texture classes, and physical parameters to represent soils associated with rain gardens. The RG soil maps were based on Ohio EPA requirements for rain gardens (Table 6), which suggest that the soil media depths of a rain garden are 60–100 cm deep with loamy sand [49]. In the updated model configurations for each implementation scenario, we assumed no underdrain pipes and no outlet pipes, which are currently not implementable in VELMA.

In addition to the soil maps, we created new land cover maps for each spatial implementation level of RG (25% to 100%). We defined a new land cover, “RG,” where existing sidewalks were located. For example, at the 50% RG implementation level, 50% of sidewalk’s pixels of original were defined as “RG” land cover. For each new “RG” map, we parameterized the PET parameters of “RG” land cover to lawn values (Table 7).

For PP scenarios, we generated soil maps with a new soil class, “PP” which replaced parking lots and driveways at each conversion level (25% to 100%). We modified the original soil depths, soil texture classes, and soil physical parameter values for the “PP” soil class (Table 6) using the same values at each conversation level. According to the Ohio EPA Stormwater Management Practices manual, the recommended thickness of a PP system is 40–76 cm, depending on frost depth [49]. Therefore, for PP, we parameterized the hydraulic conductivity and other soil physical parameter values of the first 100 cm of the “PP” soil class [32]. We assumed that permeable pavement is a continuous pavement system (gravel) and well maintained with no clogging issues.

For RB, we created soil maps with a new soil class (Table 6) and a new land cover class (Table 7), “RB” to replace current soils and land cover at 20 m and 40 m on each side of streams where agriculture exists. Because most riparian buffers for Ohio streams are forested [49], we parameterized the soil parameters and PET values of the buffer area in the new soil and cover maps to reflect the effect of a forest rooting system and forest canopy on infiltration and ET (Tables 6 and 7).

For RG, PP and RB, new permeability fraction maps were also created for each implementation level to replace the original permeability fraction map in each model scenario. In the new permeability fraction maps, permeability fractions of 0 for impervious surfaces, such as sidewalks, parking lots and driveways, were changed to 1 and 0.95 for RG and PP, respectively. The permeability fraction for the RB scenario was changed from 0.95 for agriculture to 1 for RB forested land cover.

Once the base model was calibrated, we ran the model for each of the 10 scenarios under the different LID spatial configurations to evaluate changes in peak flows, surface runoff, ET, subsurface runoff and infiltration, and compared them to that of the base model (existing conditions).

## 3. Results

### 3.1. Calibration and Validation of the Base Model

**Figure 4:**
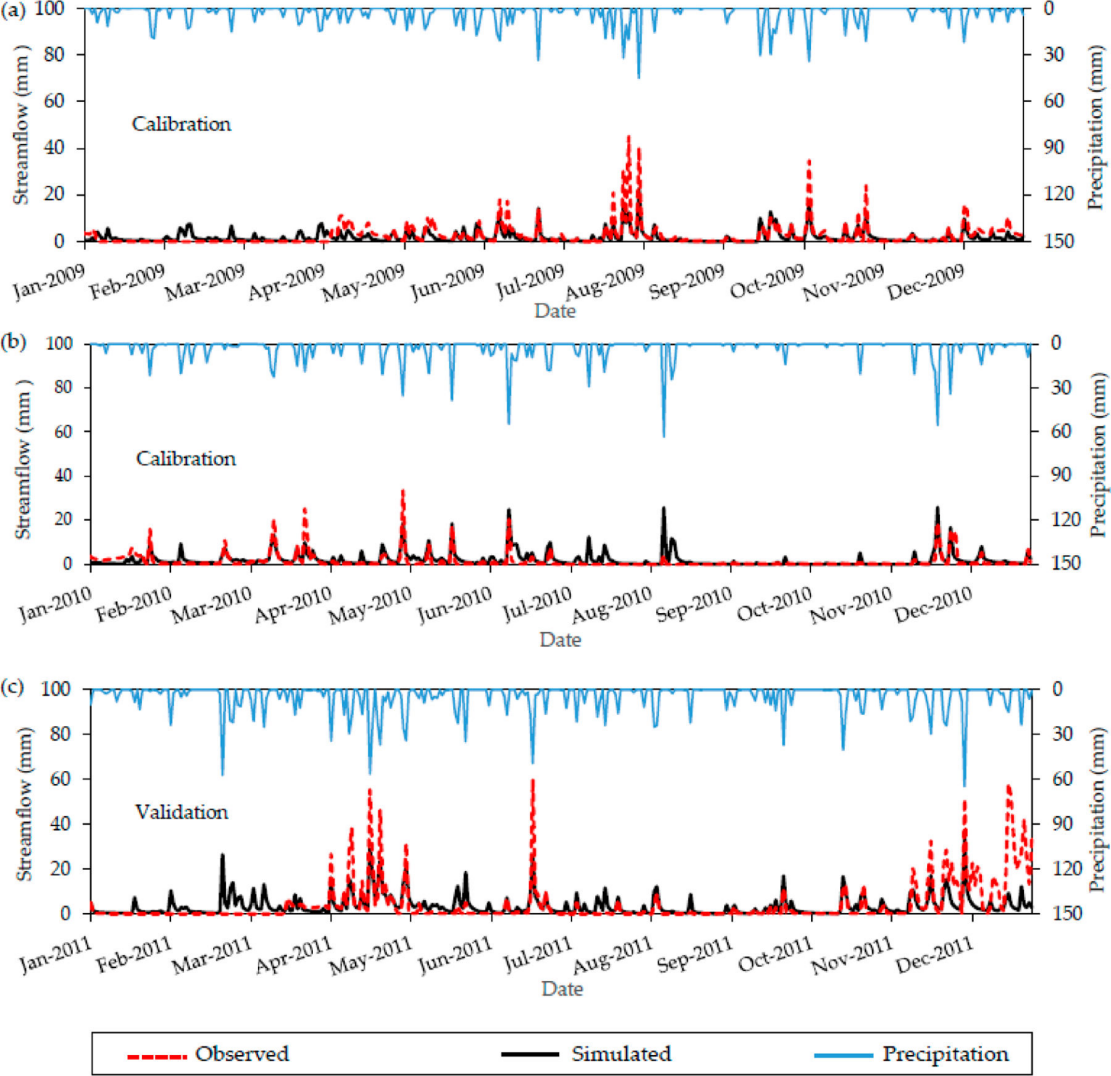
Plots of (**a**,**b**) calibration (NSE = 0.54, *R*^2^ = 0.53, RMSE = 3.12, and PBIAS = *−*2.40) and (**c**) validation (NSE = 0.40, *R*^2^ = 0.48, RMSE = 5.27, and PBIAS = 13.84) of the VELMA model output at the watershed outlet. The model was calibrated at a daily time step from 1 January 2009 to 31 December 2010 and validated at a daily time step from 1 January 2011 to 31 December 2011.

Daily streamflow calibration suggests acceptable model results across the simulation period (Figure 4a–c). The NSE, *R*^2^, root mean square error (RMSE), and percent bias (PBIAS) for the calibration period (2009 and 2010) were 0.50, 0.53, 3.12 and *−*2.40, respectively. Moriasi et al. [50] recommended that an NSE *≥* 0.50 and PBIAS *≤ ±*15 can be considered satisfactory for daily streamflow simulations. RMSE varies from 0 to large positive values. The lower the RMSE, the better the model fit [46]. The optimum value for PBIAS is zero, and low magnitude values indicate better simulations. Negative values indicate model overestimation [50]. While the daily model calibration is acceptable, it tends toward underestimating peak flows (Figure 4a,b). This is confirmed by a negative PBIAS; however, the magnitude is low, which means that the bias toward peak flow underestimation is minimal. Further, the Sim:Obs were 0.77, 1.10 and 0.96 (for 2009, 2010, 2011, respectively), all of which indicated that annual volumetric streamflow estimates in the base model were satisfactory.

Simulations during the validation period captured general daily streamflow patterns; however, the model fit was less satisfactory than the calibration period (NSE of 0.40, *R*^2^ of 0.48, RMSE of 5.27, and PBIAS of 13.84). Moreover, visual inspection of the validation plot (Figure 4c) indicated that the calibrated parameters were less successful during 2011, suggesting that calibrated model simulations may have increased limitations during wet years.

The average annual water balance components of the calibrated base model across the watershed, for the simulation period (2009–2011), are shown in Table 8. Evapotranspiration accounts for about 44% of precipitation, which approximates the lower end of the Sanford and Selnick [52] estimates of the fraction of precipitation lost to evapotranspiration in Southwest Ohio, USA.

### 3.2. LID Scenarios

**Figure 5:**
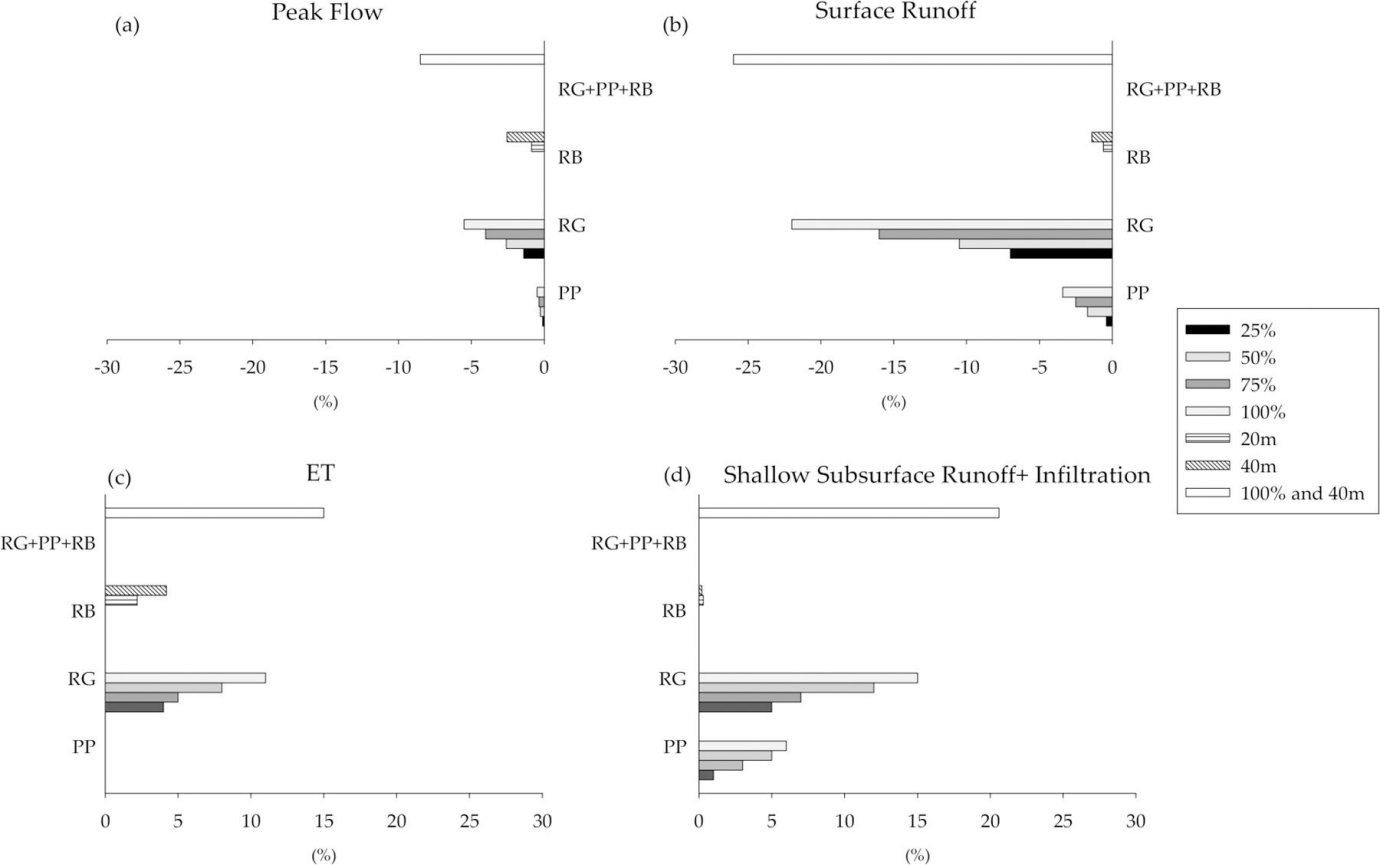
Percent change for watershed water balance components for three different types of LID practices at the outlet of the watershed (RG: Rain Garden, PP: Permeable Pavement, and RB: Riparian Buffer), (**a**) peak flow; (**b**) surface runoff; (**c**) ET (evapotranspiration); and (**d**) shallow subsurface runoff and infiltration. At the maximum level of implementation (100% and 40 m) RG, PP, and RB cover 6.4%, 2.1%, and 3% of the total watershed area, respectively.

We compared the simulated water balances for the three LID practices at 25%, 50%, 75% and 100%, 20 m and 40 m implementation levels and one combined LID scenario at the maximum level of implementation. Our results suggest that LID practices decreased surface runoff and peak flow, and increased ET, shallow subsurface runoff and infiltration as the LID implementation level increased (Figure 5). However, the response varied among different LID practices (Figure 5).

Reduction in peak flows varies from about 0.5% to 5.5% among all individual LID practice scenarios, with the high reduction observed for the RG scenario at 100% and 75% implementation levels (5.5% and 4%, respectively), followed by 50% RG and 40 m RB scenarios (Figure 5a). Surface runoff decreased across all LID scenarios, with the largest reductions resulting from the RG scenario at 25%, 50%, 75% and 100% implementation levels (7%, 10.5%, 16% and 22%, respectively) (Figure 5b). PP and RB scenarios showed smaller reductions in surface runoff, ranging from 0.4 (for 25% PP) to 3.4% (for 100% PP). Reductions in surface runoff for 40 m and 20 m RB scenarios were 1.4% and 0.6%, respectively (Figure 5b). The percentage reduction in surface runoff was more than peak flows across all scenarios.

Retrofitting the baseline model with the LID increased shallow subsurface runoff and infiltration with increasing implementation levels as shown in Figure 5c,d. ET increased 2–15% for RG and RB scenarios across implementation levels, with higher increases in the 100%, 75% and 50% RG scenarios (11%, 8% and 5%, respectively). The RG scenario resulted in higher increases for both processes in comparison to other individual scenarios. Following the same trend, PP and RB scenarios increased shallow subsurface runoff and infiltration, ranging from 0.2% to 6% for different implementation levels (Figure 5d). Changes in ET for PP scenarios were negligible (Figure 5c).

Combining the three LID practices (RG, PP, and RB) at the highest implementation levels (100% for RG and PP, and 40 m for RB) resulted in the largest reductions in peak flows and surface runoff compared to individual LID implementations. The reductions in peak flow (8.5%) were modest, but considerably greater in surface runoff (26%; Figure 5a,b). The combined LID scenario resulted in the greatest increase in ET (15%), as well as a shallow subsurface runoff and infiltration (21%), in comparison with individual LID scenarios (Figure 5c,d).

**Figure 6:**
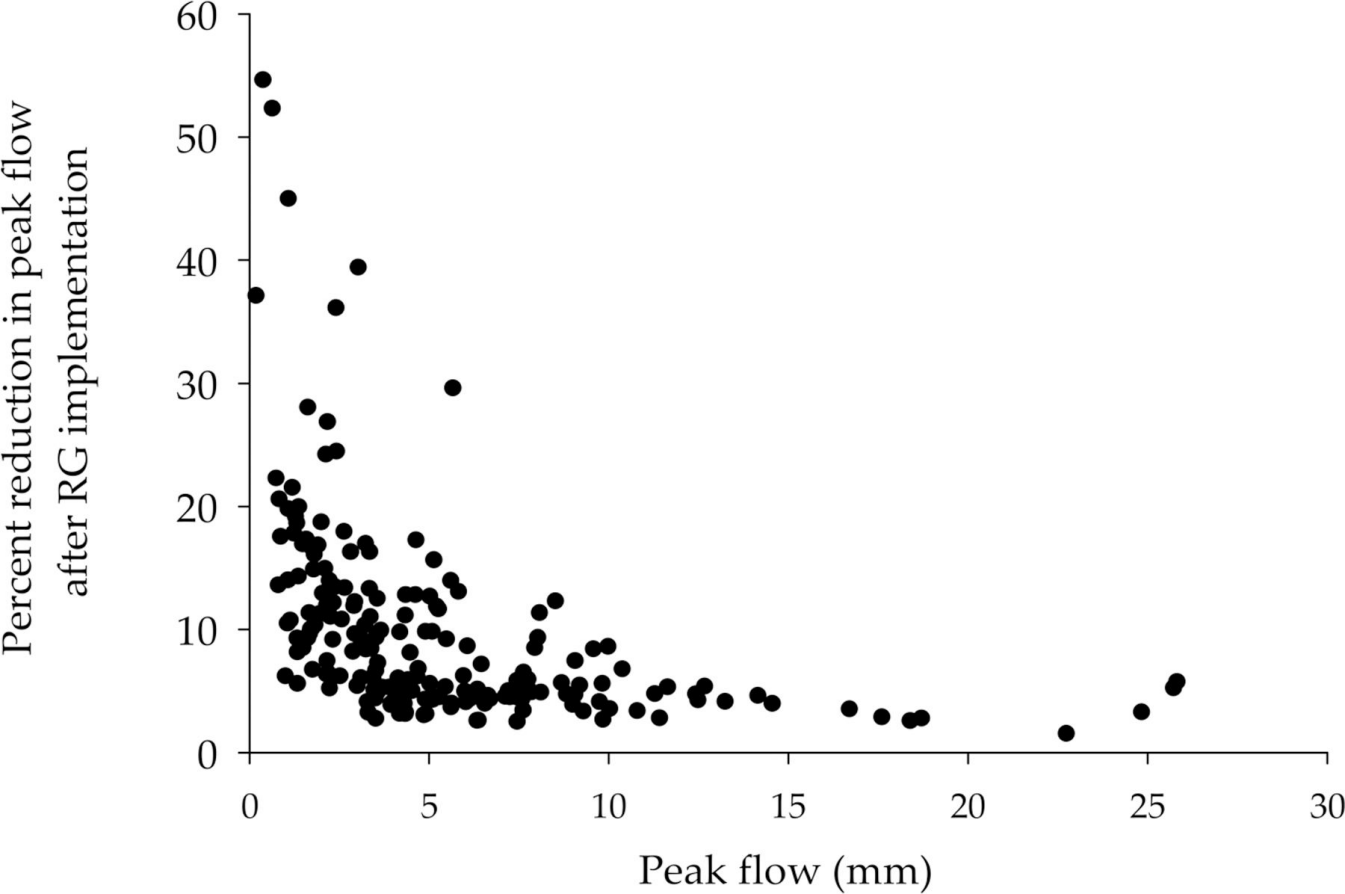
Percentage reduction in peak flow after RG implementation (100% scenario) vs. peak flow (mm) during the simulation period at the watershed outlet. Peak flow was estimated as any flow that was one standard deviation above the mean simulated daily flow for the study period or flow on the day following peak flow conditions, as described (to capture part of the falling limb of the hydrograph).

The RG scenario showed the highest reduction in peak flows in comparison with PP and RB scenarios (Figure 5a). Therefore, we compared the peak flow to the percent of reduction in peak flow after RG implementation (100% scenario) during the simulation period (Figure 6). Peak flows were defined as one standard deviation above the mean simulated daily streamflow (here, 3.18 mm). We also considered the streamflow one day after we considered the peak flows to include a portion of the falling limb of the hydrograph. The percentage reduction in peak flows after RG implementation decreased exponentially with increasing peak flow conditions *R*^2^ of 0.47; *p*-value < 0.001 (Figure 6).

## 4. Discussion

### 4.1. LID Practices and Watershed-Scale Hydrological Effects

We assess, via spatially explicit model simulations, the relative effects of different types and configurations of LID practices on watershed hydrology in a mixed land cover system. Model simulation results suggest reductions in peak flows and surface runoff, and increases in evapotranspiration and subsurface flow and infiltration, with all spatial configurations of LID at the watershed scale. This is consistent with Gagrani et al. [19], Fry and Maxwell [53], and Avellaneda et al. [54], who reported similar effects on water balance components and peak flows after the placement of different LID practices in urban watersheds, with 42–55 percent impervious surfaces and drainage areas ranging from 0.2 km^2^ to 12 km^2^.

The magnitudes of simulated water balance responses to LID placement in our watershed study were lower than other studies in strictly urban watersheds (e.g., Fry and Maxwell [53] and Avellaneda et al. [54]) and more similar to a pilot study in a small suburban watershed (1.8 km^2^) of Cincinnati, Ohio, where retrofitted rain gardens and rain barrels did not result in substantial runoff reductions [55]. The more limited response in our study watershed reflects, in part, the smaller extent of urban and suburban land cover compared to studies in other watersheds. Only 27 percent of our study watershed was covered in impervious surfaces, and only 31 percent of this area was converted to LID at the highest level of implementation. Therefore, our results are not completely unexpected and may point to important scale issues regarding the extent to which LID influences hydrologic regimes in mixed land use watersheds.

The simulated RB scenario did not result in a significant effect on peak flow at the watershed outlet and other water balance components. This is likely because only 3% of the total watershed area (and 13% of the watershed’s agricultural land cover) was converted to RB at the highest implementation level (40 m). This indicates that the type and extent of LID practices affects cumulative watershed-scale hydrological effects [15,56].

In the mixed land cover SHC watershed, model comparisons among LID scenarios suggested that the RG was most effective across all implementation levels at reducing runoff and peak flow, and promoting ET, compared to PP and RB. RG also exerted greater control over modifying watershed water balance components, in terms of per unit area LID conversions. For example, at the 100% implementation level, RG reduced peak flows by 73% and 2%, decreased surface runoff by 50% and 86%, and increased ET by ~100%, which was 23% more than PP and RB, respectively. PP was 28% more effective at increasing shallow subsurface runoff and infiltration than RG. Recent studies point to a similar effectiveness of RG on water balance components at watershed scales [16,18]. Studies also have shown that PP can effectively mitigate surface runoff [57,58]; however, the degree of RG and PP functionality depends on the extent of the application area of LID within the watershed [59].

We found that 100% implementation of RG across the watershed was more effective at reducing peak flows during small storms than during larger ones (Figure 6). At the plot scale, Speak et al. [60] found that a green roof runoff retention significantly decreased during high rainfall events. These indicate that the effectiveness of any type of management practice, including LID, may be exponentially diminished as it loses storage capacity and becomes saturated. This counters Wadzuk et al. [61], who concluded, at the plot scale, that antecedent soil moisture conditions and “back-to-back events” are not a primary concern for biofiltration rain garden and green roof practices in recovering their infiltration capacity. However, based on our results, this finding may not be transferable to watershed scales, especially when LID practices receive both precipitation and appreciable surface runoff loading [62]. Our findings also highlight the potential importance of RG in controlling the first flush of pollutant loads and channel erosion [63,64] during more frequent storm events and a need for future research on the impact of LID practices across variable sequences of wet weather events.

### 4.2. Implications for Stormwater Management and Future Research

Our study provides scientists and watershed managers a glimpse into the potential influence of LID practices in mixed land cover systems, where only a portion of the watershed is converted to LID. Watershed-scale models, such as VELMA, can provide a physically-based and systematic means of projecting and evaluating the influence of various LID configurations in heterogeneous watersheds. Using this approach, our results suggest modest to minimal changes in most components of the water balance in response to LID, though these responses may be much more considerable if the watershed was exclusively urban or suburban land cover and converted to LID.

It is important to note that the location of the LID implementation with respect to the watershed outlet may also be critical [56]. For example, in our study, the watershed in agricultural areas are in the headwaters and LID implementation is in the lower portion of the watershed. Therefore, while our results suggest a limited shift in watershed hydrological dynamics with LID implementation, if LID was implemented into the upgradient of agricultural or forested land, then the magnitude of the response may be even less substantial due to attenuation from downgradient watershed processes. Based on these results, we suggest that future research needs to evaluate the hydrological effects of LID using distributed models, with a particular focus on how configurations of different land cover types influence watershed-scale LID responses, retiming of runoff delivery from subbasins of differing land cover, and antecedent soil moisture, as affected by storm sequences.

Model selection for assessing the hydrological effects of watershed-scale LID implementation is challenging because it involves trade-offs in achieving the necessary fidelity (i.e., the extent to which the model faithfully represents the modeled system) to hydrological, biological, and biogeochemical processes for prediction accuracy, while minimizing complexity and uncertainty [14]. Careful consideration of these tradeoffs is needed for future work that addresses how LID affects watershed-scale hydrological processes, particularly in mixed land cover systems. For example, existing models that explicitly integrate LID practices have been developed for urban systems and have specific LID modules for urban-based hydrological processes (e.g., SWMM and Green Infrastructure Flexible Model (GIFMod) [65]). On the other hand, models that have been explicitly developed to assess the effects of LID practices in mixed land cover watersheds (e.g., VELMA and Regional Hydro-Ecological Simulation System (RHESSys) [66]) may have a strong biogeochemical module (because of their mixed land cover focus) but a more limited hydrological capacity to physically represent LID practices, as compared with a model such as SWMM. Responses to these challenges are evolving by incorporating LID modules within ecohydrological models, such as VELMA, that provide mechanistic representations of LID performance [14] and coupling models to quantify how LID affects the fate and transport of various pollutants, as well as couple SWMM with other watershed models to improve simulations of urban hydrology [67].

Our findings suggest a clear need for the evaluation of the influence and benefits of LID in the context of other watershed land uses and their associated management [68]. For example, the incremental influence of LID on overall watershed responses, relative to management targets at different locations along the stream network, should be assessed. In this example, if a management goal is peak attenuation at the watershed outlet, a cost-benefit analysis, of how “best” to manage diffuse sources of runoff across different land cover types for peak flow reduction, would be beneficial.

Advancing the scientific understanding of the hydrological responses to LID in mixed land cover systems and linkages with the provision of diverse benefits is imperative because of the large number of watersheds globally that have mixed land cover. Future research may focus on upscaling fine scale studies to watersheds, applying a host of hydro-ecological models with LID modules or model parameter representations to address LID challenges in suburban watersheds (e.g., to provide multiple lines of evidence to support predicted outcomes), understanding LID’s role in modifying baseflow (e.g., Bhaskar et al. [20]), and advancing these studies across diverse physiographic regions. Furthermore, given that we simulated and interpreted LID effects in this relatively small mixed land cover watershed, future research that applies continuous model simulations to project the hydrological effects of different LID configurations in mixed land cover watersheds with even greater complexity than ours will help to set realistic expectations for long term LID performance in these systems. Finally, research is also needed that expands our approaches to quantifying the effects of LID practices on nutrient and sediment loads, and links LID modules within watershed-scale ecohydrology models with simulated or measured in-stream processes.

## 5. Conclusions

We provide one of the first studies, to our knowledge, that assesses the relative watershed-scale hydrological effects of different types and configurations of LID practices in a mixed land cover watershed using a spatially explicit modeling approach. We simulated 10 scenarios across multiple spatial configurations of LID to evaluate the watershed hydrological responses of three practices—rain gardens (RG), permeable pavements (PP), and riparian buffers (RB)—in a 0.92 km^2^ watershed with mixed suburban, agricultural, and forest land cover. A spatially-explicit ecohydrological model (VELMA) was used to compare changes in the watershed’s water balance before and after LID practice implementation.

Overall, we found that the type and extent of LID practices influence watershed hydrological responses in our study system. Our simulation results indicate that LID practices decreased surface runoff and peak flow, and promoted ET, shallow subsurface runoff and infiltration. However, hydrologic responses and effectiveness varied among LID practices and implementation levels. When LID practices were considered individually, on a LID per unit area basis across all LID implementation levels, RG was more effective in reducing runoff and peak flow, and promoting ET, than PP and RB. However, our results indicated that the 100% implementation of RG was more effective at reducing peak flows during small storms than larger ones, suggesting that LID storage capacities are reduced due to soil saturation during and following large events. Further, both RG and PP increased shallow subsurface runoff and infiltration to almost the same extent at the watershed outlet and the combined LID scenario resulted in the highest performance by increasing shallow subsurface runoff and infiltration, and evapotranspiration by 21% and 15%, respectively, and reductions in peak flow and surface runoff of 8.5% and 8%, respectively.

We conclude that the spatial configurations and extent of LID practices, as well as the model selection and degree of watershed heterogeneity, might be critical for assessing the hydrological responses of watershed-scale LID implementation and must be considered in future research. Further research is needed to apply different LID configurations within mixed land cover watersheds to better understand LID performance and to evaluate the effect of LID practices on nutrient and sediment loads.

## Figures and Tables

**Table 1.** Summary of Shayler Crossing watershed land use area and characteristics.

Land Use Type	Area (km^2^)	% of Watershed	% of Total Imperviousness
Lawn	0.34	37.0	-
Agriculture (corn)	0.21	23.0	-
Forest	0.12	13.0	-
Building	0.11	12.0	44.5
Street	0.06	6.5	24.0
Sidewalk	0.06	6.4	23.5
Parking Lot and Driveway	0.02	2.1	8.0

**Table 2.** Soil parameters for the base model (* = calibrated; all other values from McKane et al. [32]). *R* stands for Rossmoyne and *A* stands for Avonburg soil type.

Parameter	Description	Soil Type	Layer	Value	Unit
z	Soil layer thickness	All	1,2,3,4	500, 500, 12,000, 12,000	mm
*K*_s,l_ *	Saturated lateral hydraulic conductivity	*R* and *A* Impervious area	1,2,3,4	130, 100, 80, 30 50, 30, 15, 10	-
*K*_s.v_ *	Saturated vertical hydraulic conductivity	*R* and *A* Impervious area	1,2,3,4	14, 14, 10, 10 7,7,5,5	-
*n*	Porosity fraction	*R* and *A* Impervious area	All	0.501 0.475	- -
*P*_b_	Bulk density	*R* and *A* Impervious area	All	1.42 1.21	g cm^−3^
θ_wp_	Wilting point	*R* and *A* Impervious area	All	0.133 0.272	- -
θ_fc_	Field capacity	*R* and *A* Impervious area	All	0.33 0.396	- -

**Table 3.** Calibrated Potential Evapotranspiration parameters for the base model.

Parameter	Description	Cover Type	Value	Unit
PetParam1	First term of PET *Hamon* equation	Agriculture (corn) Forest Lawn Impervious area	0.20 0.30 0.15 0.05	-
PetParam2	Second term of PET *Hamon* equation	Agriculture (corn) Forest Lawn Impervious area	0.622	-
TemperaturePetOff	PET is only active when air temperature is greater than this value	Agriculture (corn) Forest Lawn Impervious area	−3	C°
roair	Air density	Agriculture (corn) Forest Lawn Impervious area	1300	g m^−3^
be	ET coefficient used in the logistic equation that computes ET from PET	Agriculture (corn) Forest Lawn Impervious area	3.07	-
noTranspirationPetFraction	The fraction of PET available outside of this cover’s growing season	Agriculture (corn) Forest Lawn Impervious area	1	-

**Table 4.** LID configurations in the model and conversion levels.

Type of Current land use	Type of Spatial Configuration Maps Under LID Scenarios	Type of LID Practices	Conversion Level
Sidewalks	Soil map, cover map, and permeability fraction map	Rain Garden (RG)	25%, 50%, 75%, 100%
Parking Lots and Driveways	Soil map and permeability fraction map	Permeable Pavement (PP)	25%, 50%, 75%, 100%
Agriculture	Soil map, cover map, and permeability fraction map	Riparian Buffer (RB)	20 m and 40 m

**Table 5.** The percent of the watershed converted to each LID practice and implementation level.

Type of LID Practices	Percent Watershed Converted			
	25%	50%	75%	100%
RG	1.6	3.2	4.8	6.4
PP	0.5	1.1	1.6	2.1
	**20 m**	**40 m**	-	-
RB	1.5	3.0	-	-

**Table 6.** Soil parameter values used in the LID practice scenarios. RG: Rain Garden, PP: Permeable pavement, and RB: Riparian Buffer.

Parameter	Description	LID Practice	Layer	Value	Unit
z	Soil layer thickness^1^	All	1,2,3,4	500, 500, 12,000, 12,000	mm
*K*_s,l_	Saturated lateral hydraulic conductivity ^2^	RG PP RB	1,2,3,4 1,2,3,4 1,2,3,4	200, 200, 80, 30 250, 250, 80, 30 300, 300, 80,30	-
*K*_s.v_	Saturated vertical hydraulic conductivity ^2^	RG PP RB	1,2,3,4 1,2,3,4	50, 50, 10, 10 100, 100, 10, 10 50, 30, 15, 10	-
*n*	Porosity fraction ^2^	RG PP RB	All	0.437 0.437 0.437	-
*P*_b_	Bulk density ^2^	RG PP RB	All	1.65 1.65 1.65	g cm^−3^
θ_wp_	Wilting point ^2^	RG PP RB	All	0.055 0.033 0.055	-
θ_fc_	Field capacity ^2^	RG PP RB	All	0.125 0.091 0.125	-

1 Ohio EPA [49];

2 McKane et al. [32], based on the Ohio EPA recommended soil texture class of loamy sand and sand for RG and PP [49]. For the RB scenario, the values were set to the loamy sand soil texture class to represent a forest rooting system [32].

**Table 7.** Potential Evapotranspiration parameter values used in the LID practice scenarios. RG: Rain Garden, PP: Permeable Pavement, and RB: Riparian Buffer.

Parameter	Description	LID Practice	Value	Unit
PetParam1 ^1^	First term of PET *Hamon* equation	RG PP RB	0.15 0.05 0.30	-
PetParam2	Second term of PET *Hamon* equation	RG PP RB	0.622	-
TemperaturePetOff	PET is only active when air temperature is greater than this value	RG PP RB	−3	C°
roair	Air density	RG PP RB	1300	g m ^−3^
be	ET coefficient used in the logistic equation that computes ET from PET	RG PP RB	3.07	-
noTranspirationPetFraction	The fraction of PET available outside of this cover’s growing season	RG PP RB	1	-

1 The values for RG and RB were set to the calibrated values for lawn and forest land cover (Table 3). The value for PP set to minimum value for impervious area (Table 3).

**Table 8.** Average annual water balance components of the base model for the entire watershed, from 2009–2011. Note that precipitation is lower than the sum of the other water balance components because of the structure of VELMA’s hydrological model output, which couples shallow subsurface runoff with infiltration.

Water Balance Component	Value (mm)
Precipitation	1249
Surface runoff	444
ET	548
Shallow subsurface runoff + infiltration	427
